# Antivenom therapy: efficacy of premedication for the prevention of adverse reactions

**DOI:** 10.1186/s40409-018-0144-0

**Published:** 2018-02-28

**Authors:** Victor Morais

**Affiliations:** Department of Biotechnology, Institute of Hygiene, Faculty of Medicine, University of the Republic, Uruguay, Av. Alfredo Navarro, 3051 Montevideo, Uruguay

**Keywords:** Premedication, Snakebite accident, Adverse reactions, Hydrocortisone, Antihistaminic, Adrenaline

## Abstract

Antivenoms or antitoxins have been effectively used for more than a century. During this time, these products have always proven to be highly effective in the treatment of infections and envenomations. However, antivenoms did not exhibit good safety results in their initial applications. After many improvements, antivenoms have substantially better safety profiles but still have some side effects. Due to the occurrence of adverse reactions, the practice of using premedication with the intent to decrease side effects has become accepted or mandatory in many countries. The drugs used for premedication belong to the histamine H1 antagonist, glucocorticoid and catecholamine groups. Currently, this practice is being questioned due to low or controversial efficacies in clinical assays. In this article, we discuss the causes of adverse reactions, the mechanisms of drugs that block the undesired effects and the results obtained in clinical trials. Although these three families of drugs could have positive effects on reducing adverse reactions, only adrenaline has demonstrated positive results in clinical assays.

## Background

Heterologous neutralizing serums, usually called antivenoms, antiserums or antitoxins, consist of neutralizing antibodies produced in animals (mainly horses and sheep) and have been effectively used for more than a century [1–3]. In 1890, von Behring and Kitasato demonstrated that the serum of a diphtheria-infected animal confers immunity against the same disease to naive animals [3, 4]. Some years later, antiserum began to be used in humans. Since that time, such products have always proven to be highly effective in the treatment of infections and envenomations [1, 4–7]. However, in their initial applications, antivenoms did not exhibit good safety results and could even cause life-threatening side effects [8]. The main reason was that first antivenoms were poorly purified preparations or crude sera. Over the years, for many of the original applications, heterologous serums were replaced by other drugs with better safety profiles, such as antibiotics, vaccines and homologous serums. However, in cases of envenomation by snakes, scorpions or arachnids, antivenoms remain the only effective treatment [4]. Currently, after many improvements, antivenoms exhibit acceptable safety profiles [1, 9, 10]. Nevertheless, antivenom quality still varies widely depending on the producer, while some antivenoms exhibit adverse reaction rates of less than 10%, others have values of greater than 50% [11, 12].

Due to these variations, recently the Brazilian National Health Surveillance Agency (ANVISA, a regulatory agency) launched the resolution RDC No. 187 on November 8th, 2017 [13]. It establishes the minimum requirements for the registration of antivenoms in order to guarantee the quality, safety and efficacy of these products. Two points are relevant: first, non-clinical studies designed with the objective of determining at least the ED50 and the power of the source material; and, second, clinical trials covering aspects of safety and efficacy. According to this new ANVISA resolution, a Brazilian group of researchers who developed the first apilic antivenom to treat massive Africanised honeybee attack, prepared a protocol to clinical trial evaluating safety and antivenom dosage [14]. At the end of this trial phase I/II, it will be possible to evaluate the adverse reactions and stablish the safety of this new antivenom.

Due to the occurrence of adverse reactions, the practice of using premedication has been accepted or is mandatory in many countries. The most commonly used drugs are corticoids, antihistamines and, more rarely, adrenaline [12, 15]. Currently, this practice is being questioned due to low or controversial efficacy [12].

In the present work, we studied the mechanisms of action of antivenom-induced adverse reactions and premedication drugs, the mechanisms of interference with the side effects elicited by these drugs, and the correlations of the possible mechanisms of action and clinical observations.

## Mechanism of adverse reactions

The adverse reactions elicited by antiserums are shown in Table 1 and can be classified into early adverse reactions and late adverse reactions. Early adverse reactions occur within 24 h of the administration of antivenoms and are the most severe [11]. Late adverse reactions, traditionally known as “serum sickness”, occur from 5 until 20 days after antivenom administration [11].

**Table 1:** Types of adverse reactions caused by antivenoms

Adverse reaction	Type	Cause	Mechanism	Main physiological effects
IgE-mediated anaphylactic	Early	Presence of patient IgE against any component of antivenom	Basophil and mast cell degranulation by IgE. Release of histamine, prostaglandins, leukotrienes and other pharmacological mediators	Increased vascular permeability, vasodilatation, bronchial and visceral smooth-muscle contraction, anaphylactic shock
Non IgE-mediated anaphylactic	Early	Presence of aggregates, Fc fragments or heterophilic antibodies against blood cells in antivenom	Complement activation by Ig aggregates and others. Basophil and mast cell degranulation by complement. Release of histamine, prostaglandins, leukotrienes and other pharmacological mediators	Increased vascular permeability, vasodilatation, bronchial and visceral smooth-muscle contraction, rash, urticaria, pain
Pyrogenic	Early	Presence of endotoxins in antivenom	Macrophage and other cell activation by endotoxins. TNF-α, IL-1, IL-6 production	Fever
Serum sickness	Late	Humoral immune response to antivenom	Complement activation by immunocomplex. Basophil and mast cell degranulation by complement. Release of histamine, prostaglandins, leukotrienes and other pharmacological mediators	Rash, glomerulonephritis

*Ig* immunoglobulin, *IgE* immunoglobulin E, *TNF-α* tumor necrosis factor alpha, *IL-1* interleukin 1, *IL-6* interleukin 6

### IgE-mediated anaphylactic reactions (type I hypersensitivity, immediate hypersensitivity)

Anaphylactic reactions are early adverse reactions that are mediated by IgE antibodies against any component of the antivenom. These antibodies are found attached to basophils or mast cells Fc receptors (FcεR). When specific antigens are recognized by IgE, they can produce crosslinking of the cell-bound antibodies and, in the first stage, induce the degranulation and release of active compounds, mainly histamine, prostaglandins, leukotrienes and other pharmacological mediators. These compounds lead to several actions including increased vascular permeability, vasodilatation, bronchial and visceral smooth-muscle contraction, mucous secretion and local inflammation [16]. The systemic presence of antigens, such as those that exist in heterologous antivenoms, can provoke anaphylactic shock, which is characterized by edema in several tissues and a decrease in blood pressure secondary to vasodilatation [12, 17]. This response usually occurs in patients who have previously been sensitized to some antivenom component. This response is the most severe and life-threatening adverse reaction, but occurs infrequently.

### Non IgE-mediated anaphylactic reactions (anaphylactoid reactions)

Anaphylotoxins (C3a, C4a and C5a) are low-molecular-weight active peptides that are produced by complement system activation. Anaphylotoxins stem from C3, C4, and C5 serum complement proteins and are created by the cleavage of these proteins during complement fixation by antigen-antibody complexes, immunoglobulin aggregates and other compounds [18]. In the case of antivenom, the activation of the classical way of complement mediated immunoglobulin aggregates, is probably the main mechanism involved in anaphylotoxins generation [11, 19]. Moreover, the presence of heterophilic antibodies in antivenom against human erythrocytes, neutrophils and others cell types could also contribute to generate anaphylotoxins [11]. The C5a, C3a and C4a fragments stimulate chemotaxis, neutrophil activation and the degranulation of basophils and mast cells, which release pharmacologically active mediators of immediate hypersensitivity [17]. The net effects of these activities include the contraction of vascular smooth muscle, increased vascular permeability and the migration of neutrophils and monocytes from the blood vessels [16].

Non IgE-mediated anaphylactic reactions constitute the majority of early reactions induced by antivenoms. These reactions occur in patients who have not been previously sensitized to antivenom components [11]. According to Squaiella-Baptistão et al. [20] various antivenoms from different producers are able to activate the classical pathway of the complement system and generate anaphylatoxins. These observations suggest that factors, such as composition, contaminant proteins, and aggregates, may influence the anticomplementary activity of antivenoms. Additionally, an independent mast cell activation triggered by non-complement activation has also been proposed [21].

### Pyrogenic reactions

Endotoxin contamination is the main cause of pyrogenic reactions elicited by antivenoms. Fortunately, most production laboratories implement or are beginning to implement strict quality requirements for their facilities, raw materials, processing systems and equipment to avoid endotoxin contamination, which has resulted in an important decrease in this type of adverse reaction in recent years. Bacterial endotoxins consist of lipopolysaccharides (LPS), which are major components of the outer cell membranes of gram-negative bacteria [22]. The molecular mechanism of toxicity is related to the interaction with Toll-like receptor 4 (TLR4) and/or LPS-binding protein (LPB) receptors located on monocytes and other cell components of the immune system that produce TNF-α, IL-6, interleukin 1β (IL-1β) and other cytokines [18]. Higher levels of endotoxins are related to bacterial infection or digestive tract injuries, but contamination at low concentrations can be found in pharmaceutical products. The presence of low levels of endotoxins in antivenoms generates an important increase in the frequency of mild reactions (mainly fever) in patients [9]. Finally, according to Gutierrez et al. [23], the preclinical assessment of antivenoms regarding the concept of the 3Rs (replacement, reduction, and refinement) are necessary to avoid adverse reactions in patients, especially contamination by microorganisms.

### Late adverse reactions, type III hypersensitivity (serum sickness)

This type of adverse reaction was first reported by Pirquet and Schick in 1905 [8]. These authors studied the side effects caused by the administration of large quantities of antitoxins and found that many days after antitoxin administration, some patients exhibited fever and rashes, and some reported kidney damage with proteinuria and lymphadenopathy. These authors also found that symptoms appeared more rapidly after a second exposure to the foreign serum than after the first administration.

Type III hypersensitivity is mediated by antigen-antibody complexes. As a consequence of antivenom administration, the patient’s immune system reacts by producing antibodies that attach to the antivenom, which results in the formation of immune complexes [18, 24]. These complexes lead to complement activation and leukocyte infiltration, i.e., the so-called “serum sickness” syndrome. The classic reaction occurs 7 to 15 days after the triggering injection, but manifestations can appear a few days following the injection in the accelerated form of serum sickness, which can occur in subjects who are already sensitized. The incidence of this type of reaction has not been clearly quantified because the symptoms are generally mild and occur when the patient has already been discharged; therefore, no medical record is generated.

## Action of premedication drugs

### Antihistamines

Histamine is formed by the decarboxylation of the amino acid L-histidine and is an important mediator of immediate allergic and inflammatory reactions, but it plays only a modest role in anaphylaxis. Most tissue histamine is found in the granules of mast cells and basophils [25].

The main actions triggered by histamine include the induction of edema, direct vasodilator action on arterioles and precapillary sphincters, decrease in systolic and diastolic blood pressure, increase in heart rate, stimulation of the sensory nerve endings, especially those mediating pain and itching, and bronchoconstriction in patients with asthma [25].

Additionally, histamine exhibits an active chemotactic attraction for immune cells (neutrophils, eosinophils, basophils, monocytes and lymphocytes), which, due to the vasodilation effect, cause the leakage of plasma containing mediators of acute inflammation (complement proteins and C-reactive protein) and antibodies [25].

Histamine exerts its biologic actions via an interaction with specific cellular receptors that are located on the membrane surface. The four different histamine receptors are designated H1-H4 [26]. The H1 receptor is involved in immune responses.

H1 antihistaminic agents are used to prevent or treat the symptoms of allergic reactions. Histamine is the primary mediator of urticaria, and H1 antagonists are the drugs of choice for its treatment and are also effective if administered before exposure. However, in other pathologies, such as bronchial asthma, which involves several mediators, H1 antagonists are ineffective. H1 antagonists are divided into first- and second-generation agents. Both reduce or block the actions of histamine by reversible competitive binding to the H1 receptor [26].

In antivenom treatment, promethazine and chlorpheniramine, which are both first-generation agents, are most frequently used as premedications [15]. From a theoretical perspective, antihistamine premedication can block or reduce the undesirable effects of histamine, but has no influence on the effects of other mediators such as prostaglandins and leukotrienes.

### Glucocorticoids

Glucocorticoids have widespread effects because they influence the functions of many cells and biochemical pathways in the body [27]. Such influence can have important consequences that are related to the undesirable effects of this type of medication. Most of the effects of glucocorticoids are mediated by widely distributed glucocorticoid receptors. These receptors regulate the transcription of target genes that have broad effects on the regulation of growth factors, proinflammatory cytokines, and other factors [27].

Regarding their immunological effects, glucocorticoids dramatically reduce the manifestations of inflammation. Some of the mechanisms of this process include inhibition of phospholipase A and cyclooxygenase activity and the prevention of biosynthesis of inflammatory and immune mediators. Glucocorticoids inhibit phospholipase A by inducing increased synthesis of an intracellular mediator called annexin-1 [26, 28]. Other immunosuppressant effects include reduction in the size and substance of lymph nodes and the spleen, the inhibition of helper T cells, the decrease of antibody and cytokine production, the diminishment of neutrophil and macrophage phagocytic activity and the stabilization of mast cell membranes, which reduces the amount of histamine released by basophils and mast cells. Glucocorticoids also alter the normal distribution of immune cells; the concentration of neutrophils in the circulation increases, whereas the levels of lymphocytes (T and B cells), monocytes, eosinophils, and basophils decrease [27]. Additionally, the natural glucocorticoids hydrocortisone and cortisone have mineralocorticoid activities. For this reason, glucocorticoids are important agents in the treatment of many inflammatory, immunologic and hematologic disorders [27].

Recently, Santos-Barreto et al. [29] experimentally studied the combination of antivenom and dexamethasone and concluded that the use of this glucocorticoid as an adjunct to the antivenom therapy could be useful to improve the treatment of local symptoms observed in *Bothrops* envenomation.

Glucocorticoids are classified according to their duration of action (short-, intermediate-, and long-acting forms) [26]. Hydrocortisone is a natural short-acting glucocorticoid that is widely used as a premedication in antivenom treatment [12].

The inhibition of phospholipase A and cyclooxygenase and mast cell membrane stabilization mediated by glucocorticoids should exhibit activities relevant to the prevention of anaphylactic reactions. The reduction in antibody production should also contribute to reducing late adverse reactions. Unfortunately, many other immunosuppressant effects of glucocorticoids require more time to act and turn it ineffective as a prophylactic drug against early adverse reactions [12].

### Catecholamines

Adrenaline (epinephrine) is the most widely used catecholamine drug for the prevention and/or treatment of early adverse reactions to antivenoms. Unlike antihistamines and glucocorticoids, adrenaline does not interfere with the mechanisms of adverse reactions. Adrenaline exhibits strong actions that directly oppose the effects triggered by mast cell and basophil activation. It is an agonist at both α and β adrenoceptors resulting in a potent vasoconstrictor and cardiac stimulant. The α1 receptors are widely expressed in vascular beds, and their activation leads to arterial and venous vasoconstriction. The stimulation of β receptors in the heart increases cardiac output. The activation of β2 receptors in the bronchial smooth muscle leads to bronchodilation [30]. Adrenaline also has other activities in many organs and tissues, including eyes, genitourinary organs, salivary glands, apocrine sweat glands, fat cells, liver, pancreatic islets and other endocrine glands [30].

Due to the strong and extensive actions of adrenaline, many hospitals prefer to use it only for the treatment of acute adverse reactions and not for pretreatment [12, 15]. The syndrome composed of bronchospasms, mucous membrane congestion, angioedema, and severe hypotension observed in anaphylactic shocks usually responds rapidly to the parenteral administration of adrenaline [30].

## Clinical assays

Between 1989 and 1993, Bucaretchi et al. [31] studied in an observational clinical study the type and frequency of adverse reactions in 24 children who received pretreatment with H1 and H2 antihistamines and glucocorticoids. These authors found an overall early adverse reaction rate of 33% and suggested that pretreatments did not exhibit any protective effect [31].

Fan et al. [32] investigated in a sequential randomized, double blind, placebo controlled trial, the efficacy of an antihistamine (promethazine) in the prevention of early reactions to horse antivenom administration in the Vital Brazil Hospital, Butantan Institute. The authors recruit 101 patients from 1994 to 1995 and found no significant differences between patients who received promethazine and those who did not, in terms of the occurrence of early reactions. The reactions were mild to moderate and occurred in 24% of patients treated with promethazine and 25% of those who received placebo.

In a retrospective observational clinical study from 1994 to 2004, Williams et al. [33] examined antivenom use, premedication and early adverse reactions in patients after snake bites in 11 rural health facilities in Papua New Guinea (136 antivenom documented cases). These authors found adverse reaction rates of 28% in unpremedicated patients, 28% in premedicated patients without adrenaline and 8% in adrenaline-premedicated patients. They concluded that premedication with promethazine and/or hydrocortisone without adrenaline did not reduce early adverse reactions [33].

Similarly, Premawardhena et al. [34] in a prospective, double blind, randomized, placebo controlled trial, found beneficial effects of adrenaline administered subcutaneously immediately before the administration of antivenom. The assay was conducted between 1998 and 1999, and analyzed 105 cases. Patients who received adrenaline exhibited a decrease in adverse reactions to a rate of 11%, compared to the rate 43% observed in control patients [34].

On the other hand, in an Australian nested prospective cohort study conducted from 2002 to 2007, the authors found only a marginal decrease in adverse reactions with pretreatment drugs. They studied 195 patients and found a reduction from 23% to 18% of hypersensitivity reactions, with the use of adrenaline and no reductions with any other drug. These authors concluded that the use of premedication was not associated with any reduction in adverse reactions [35].

Between 2005 and 2008, in an extensive (1007 patients), randomized, double-blind, placebo-controlled trial in Sri Lanka, De Silva et al. [15] investigated the efficacy of promethazine, hydrocortisone and adrenaline. They found no decrease in adverse reactions with the use of promethazine or hydrocortisone. However, these authors found that pretreatment with low-dose adrenaline reduced the risk of acute severe reactions to snake antivenom by 43%. Additionally, co-administration with hydrocortisone counteracted the benefit observed with adrenaline alone. Recently, these authors published a review regarding the prevention and treatment of adverse reactions and found that only adrenaline had any reliable reports of decreasing the number of adverse reactions [12]. Other review articles have reached similar conclusions [36, 37].

In a randomized controlled clinical trial conducted in Sri Lanka in 2016, Kularatne et al. [38] tested the effectiveness of intravenous hydrocortisone in reducing adverse reactions to antivenom in 236 patients. Patients received randomly intravenous hydrocortisone at least 2 h prior to antivenom administration or received the same dose at the same time as the antivenom administration. The results revealed that hydrocortisone did not reduce the rate of adverse reactions when administered simultaneously (35%) or up to 4 h prior to the antivenom (39%). Although the authors did not have an untreated group to ascertain the efficacy of hydrocortisone, they reached the important conclusion that hydrocortisone treatment did not justify a delay in the administration of antivenom.

In contrast, two clinical assays seem to show the efficacy of hydrocortisone with an antihistamine. Gawarammana et al. [39] investigated the efficacy of infusion of hydrocortisone with or without chlorpheniramine in a prospective, double-blind, randomized, placebo-controlled trial in Sri Lanka. The trial recruited 52 patients and authors found an overall high level of adverse reactions (81% in the placebo group). Hydrocortisone infusion alone was ineffective in reducing the occurrence of acute adverse reactions, but in combination with chlorpheniramine, hydrocortisone elicited a slight but significant decrease in adverse reactions (52%). Unfortunately, the study showed an unusual high level of adverse reactions and did not investigate further the efficacy of chlorpheniramine alone.

In another study carried out in a rural mission hospital in Ecuador from 2002 to 2006, snakebite victims received a new antivenom regimen that included prophylactic drugs (hydrocortisone and diphenhydramine) with a slow intravenous infusion of diluted antivenom. The authors compared their observations to a historical control without prophylactic drugs and to the fast intravenous injection of undiluted antivenom. They found that premedication with intravenous hydrocortisone and diphenhydramine together with the intravenous administration of diluted antivenom over 60 min reduced the frequency of adverse reactions from 47% to 2% and reduced the severity of anaphylactic reactions [40]. Although the infusion rate seems to have no effect on adverse reactions, it is not possible to determine whether the beneficial effects were due to the slow administration of diluted antivenom or premedication [41, 42]. Moreover, the use of historical controls is not a robust manner to compare with a prospective study group.

## Conclusion

Clinical assays have produced a variety of results probably due to the heterogeneity of the design and the quality of the trials [43]. In addition to this, there is also a great variation in the medical services and in the quality of the antivenom, which generates an even greater degree of variability. In accordance with this, ANVISA launched a new resolution that establishes the minimum requirements for the registration of antivenoms in order to guarantee the quality, safety and efficacy of these products [13].

Despite these many difficulties, the clinical observations seem to confirm the lack of efficacy of antihistamines alone and the lack or minor efficacy of hydrocortisone in the prevention of adverse reactions. In contrast, there is evidence of the efficacy of adrenaline in decreasing adverse reactions (Table 2). However, due the potential adverse effects, many facilities prefer to use adrenaline for treatment only [12]. Further clinical assays will be necessary to confirm the real or the lack of effectivity of pretreatments in antivenom therapy.

**Table 2:** Premedication used in antivenom treatment

Drug	Possible adverse reaction	Mechanism Involved	Clinical trial efficacy
Adrenaline	Anaphylactic Non IgE anaphylactic	Strong actions that directly oppose the effects triggered by mast cell and basophil activation. Vasoconstrictor and cardiac stimulant.	Yes
Glucocorticoids	Anaphylactic Non IgE anaphylactic Serum sickness Pyrogenic	Phospholipase A and cyclooxygenase inhibition. Mast cell membrane stabilization. General immunosuppressant effects	No^a^
Antihistamines	Non IgE anaphylactic	Reduce or block the actions of histamine by reversible competitive binding to the H1 receptor	No^a^

^a^Glucocorticoids and antihistamines alone did not show efficacy but two trials show some efficacy using hydrocortisone together with an antihistamine

## Abbreviations

ANVISA: Brazilian National Health Surveillance Agency
FcεR: Immunoglobulin E Fc receptor
Ig: Immunoglobulin
IgE: Immunoglobulin E
IL-1: Interleukin 1
IL-1β: Interleukin 1β
IL-6: Interleukin 6
LPB: LPS-binding protein
LPS: Lipopolysaccharides
TLR4: Toll-like receptor 4
TNF-α: Tumor necrosis factor alpha
